# Evolution of the fruit endocarp: molecular mechanisms underlying adaptations in seed protection and dispersal strategies

**DOI:** 10.3389/fpls.2014.00284

**Published:** 2014-06-25

**Authors:** Chris Dardick, Ann M. Callahan

**Affiliations:** Appalachian Fruit Research Station, United States Department of Agriculture – Agricultural Research ServiceKearneysville, WV, USA

**Keywords:** fruit development, endocarp, dehiscence, lignification, fruit evolution

## Abstract

Plant evolution is largely driven by adaptations in seed protection and dispersal strategies that allow diversification into new niches. This is evident by the tremendous variation in flowering and fruiting structures present both across and within different plant lineages. Within a single plant family a staggering variety of fruit types can be found such as fleshy fruits including berries, pomes, and drupes and dry fruit structures like achenes, capsules, and follicles. What are the evolutionary mechanisms that enable such dramatic shifts to occur in a relatively short period of time? This remains a fundamental question of plant biology today. On the surface it seems that these extreme differences in form and function must be the consequence of very different developmental programs that require unique sets of genes. Yet as we begin to decipher the molecular and genetic basis underlying fruit form it is becoming apparent that simple genetic changes in key developmental regulatory genes can have profound anatomical effects. In this review, we discuss recent advances in understanding the molecular mechanisms of fruit endocarp tissue differentiation that have contributed to species diversification within three plant lineages.

## INTRODUCTION

**FIGURE 1:**
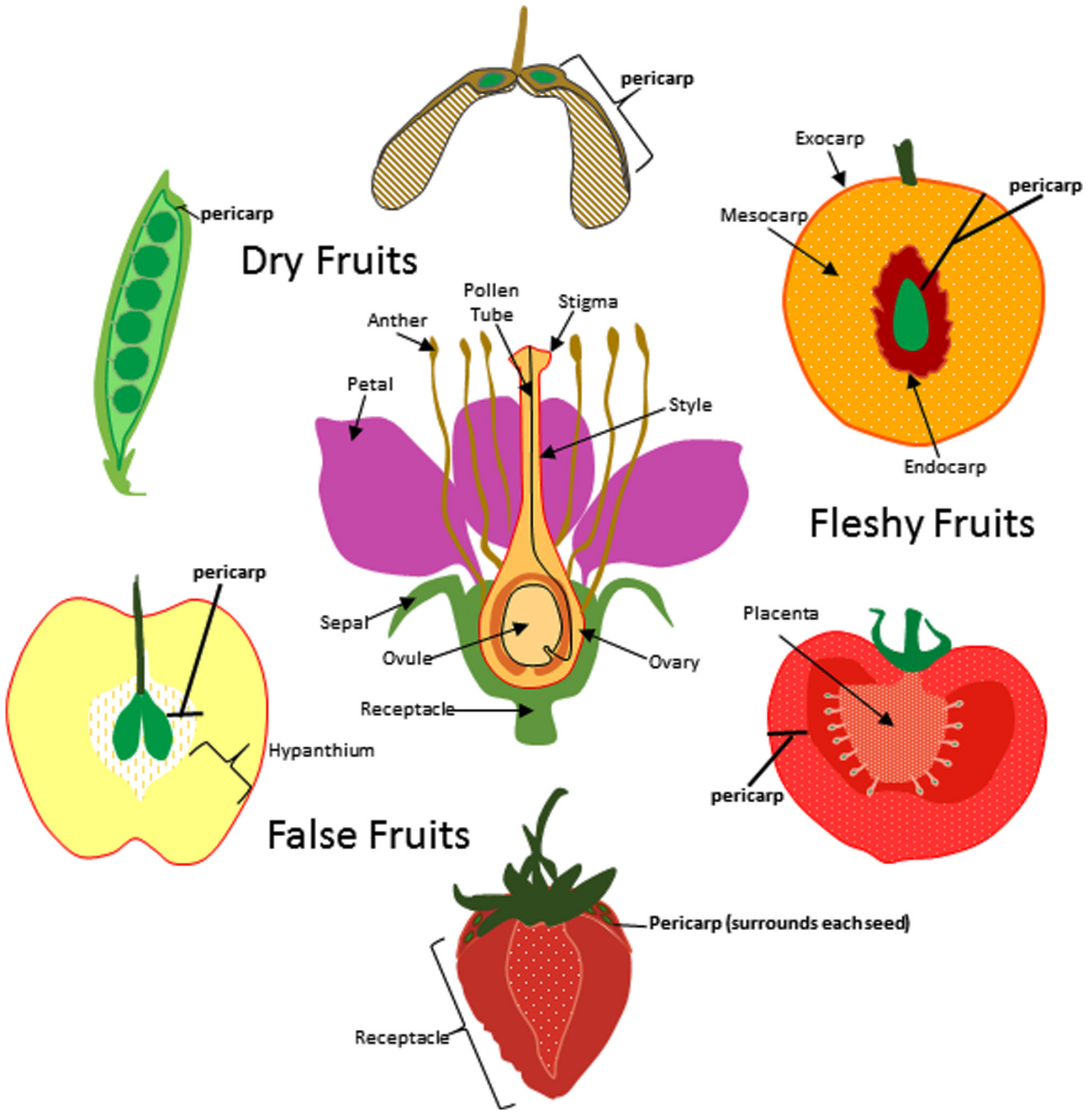
**Origin of fruit tissue layers in dry, fleshy, and false fruits.** For simplicity a flower with a single carpel is shown at center though it is important to note that many of the flowers that give rise to the fruits depicted here produce multiple carpels. The ovary and other floral tissues are indicated and the carpel is outlined in red. Pericarp (bold) is indicated for pea, maple, peach, tomato, strawberry and apple fruits. Exocarp, mesocarp, and endocarp are indicated for peach.

In general, fruits can be divided into two classes; dry fruits and fleshy fruits. Dry fruits are thought to predate their fleshy counterparts and are typically dispersed by physical forces (Scutt et al., 2006). Once the seeds mature, they are ejected by pod shattering, swept up by the wind, or adhere to animal surfaces for transport (epizoochory). In contrast, seed dispersal in fleshy fruits most often depends on animals consuming the fruit and dispersing the seeds after ingesting or discarding them. Whether it is a dry or fleshy fruit, all fruits contain tissue layers derived from the carpel ovary which are collectively called the pericarp (**Figure 1**). The pericarp can often be further differentiated into additional layers called endocarp (innermost layer), mesocarp (intermediate layer), and exocarp (skin or surface layer). Pericarp differentiation in dry fruits is often difficult to discern as each layer sometimes only contains a few rows of cells. In most fleshy fruits, the mesocarp comprises the soft edible portion of the fruit but in some exceptions the fleshy portion is formed from tissues other than the ovary (**Figure 1**). These are sometimes known as false fruits. For example, apple produces a pome fruit in which the core represents the true ovary derived fruit and the edible portion originates from the hypanthium; formed from the fused base of petals and sepals. In contrast, the fleshy portion of the strawberry is formed from the flower receptacle.

**FIGURE 2:**
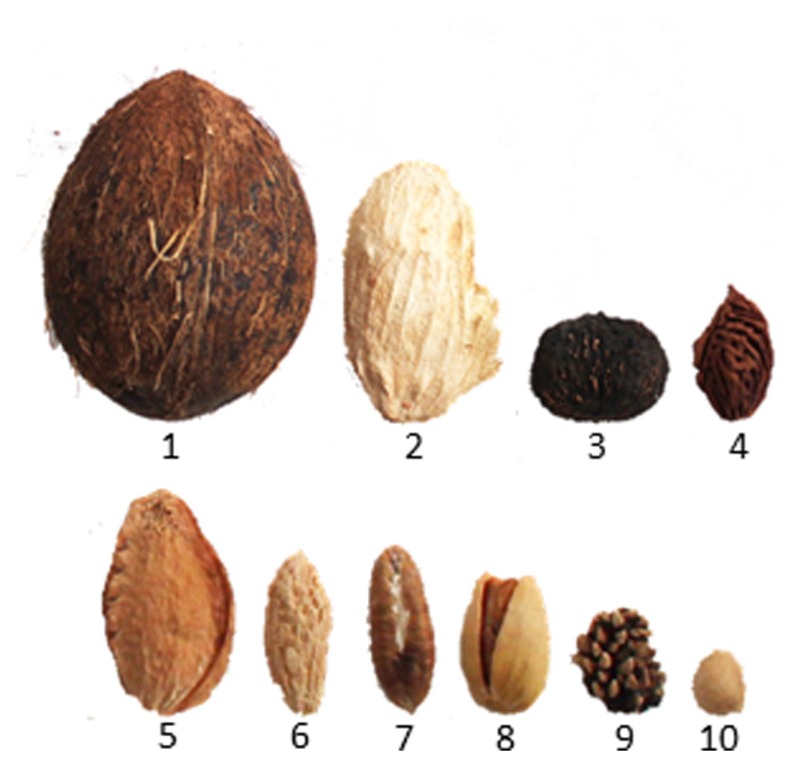
**Examples of lignified endocarps in drupes after removal of exocarp and mesocarp.** Seeds are contained inside and not shown (lower row is magnified for visibility). (1) Coconut, (2) mango, (3) Walnut, (4) Peach, (5) Apricot, (6) Olive, (7) Date, (8) Pistachio, (9) Blackberry, and (10) Cherry.

The endocarp is differentiated from the inner layer of the ovary and is the tissue layer immediately adjacent to the seed. It plays diverse roles in fruit function and can be fleshy as found in watermelon, fibrous like in mango, or extremely hard and durable as in a peach. Fruits with a hardened endocarp are called drupes. Drupes include a number of economically important crops such as peach, cherry, plum, almond, coffee, mango, olive, coconut, pistachio, date, raspberry, oil palm, and walnuts (**Figure 2**). The hardened endocarp provides a physical barrier around the seed protecting it from disease and herbivory (Doster and Michailides, 1999). The seeds of drupes are dispersed by animals either after consumption (blackberries) or upon being discarded (peaches). Once dispersed the seeds escape their woody enclosure via cracking and splitting of the endocarp shell due to environmental exposure.

In dry fruits the endocarp plays a primary role in seed dispersal. Dry fruits are generally categorized as either dehiscent or indehiscent depending on whether or not the pericarp splits open at maturity. Dehiscence is a mechanism of seed dispersal whereby the pod is forcibly opened by internal physical tension which builds during fruit maturation, causing the seeds to be suddenly discharged. Wisteria represents an extreme case in which the pods are explosive, ejecting the seeds very long distances. Other examples of dehiscent fruits include sweet pea, soybean, alfalfa, milkweed, mustard, cabbage, and poppy. Dry indehiscent fruits do not undergo this process and include a number of nuts, sunflowers, and windborn seed types such as the winged seeds found in maple and ash or cypsela-type structures produced by dandelions.

**FIGURE 3:**
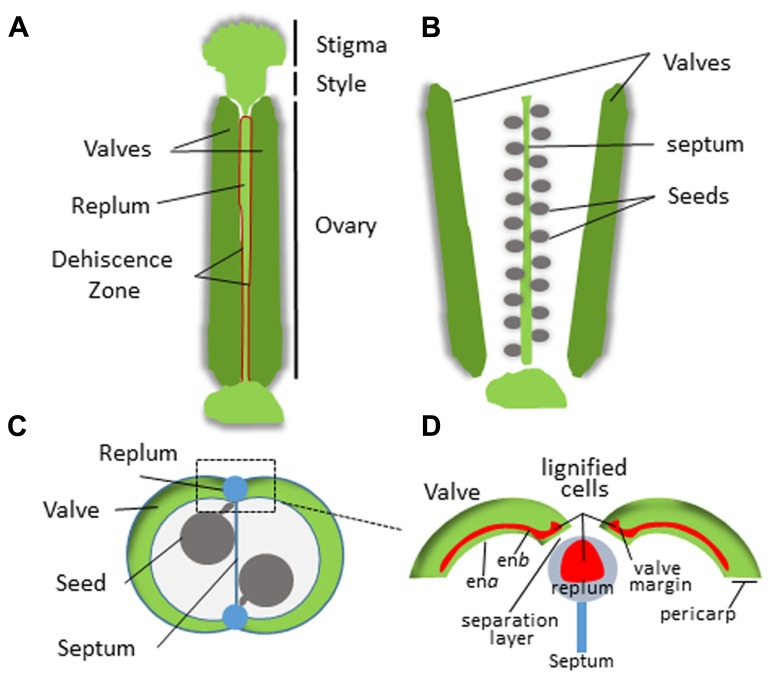
**Structure of the *Arabidopsis* silique. (A)** Intact silique prior to dehiscence. Dehiscence zone is highlighted in red. **(B)** Valve separation after dehiscence revealing the seeds attached to the septum. **(C)** Cross section of the silique. **(D)** Magnified view of the replum region. The pericarp tissue is indicated and lignification zones are shown in red.

In dehiscent fruits, differentiation of the endocarp and specialized adjoining tissue layers from the mesocarp regulates pod shatter. This process has been extensively studied in *Arabidopsis thaliana* which is in the family *Brassicaceae* (reviewed by Ferrándiz, 2002; Dinneny and Yanofsky, 2004; Dinneny et al., 2005; Lewis et al., 2006). *Arabidopsis* fruits form as a bivalved silique containing regularly arranged seeds (**Figure 3**). The pericarp in each silique forms two valves that sandwich a thin papery tissue called the septum onto which the seeds are attached. The valves are connected to the septum on two sides by an external part of the septum called the replum. The endocarp is sub-divided into two layers; endocarp A (en*a*) and endocarp B (en*b*) that line the inner surface of the valves. A distinct tissue layer referred to as the valve margin forms a hinge on either side of the replum at the tip of the silique. Upon maturation, cells within the en*a* layer secrete cell wall degrading enzymes while cells within the en*b* layer, vascular bundles within the replum, the valve margins, and patches of neighboring mesocarp lignify and harden (Ferrándiz, 2002; Liljegren et al., 2004; Ogawa et al., 2009). This simultaneous separation and hardening of the en*b,* valve margin, inner replum, and adjoining mesocarp tissues creates tension forces that eventually cause the pod to split open along a distinct separation layer that divides the valve margins from the replum. Silique dehiscence is a highly coordinated process that is tightly coupled to complex differential pericarp tissue patterning of the mesocarp, endocarp, valve, valve margins, separation layer, and replum.

In addition to seed protection and dispersal, the endocarp also plays an important role in sustaining and communicating with developing seeds. Seeds are connected to the maternal fruit tissue via an umbilical structure called the funiculus. The funiculus initiates from the seed coat and attaches to the placenta on the ovary wall. As the fruit matures, the placental layer of the ovary often becomes part of, or is fused to the endocarp.

A hallmark of both drupes and dehiscent fruits is the hardening of the endocarp as the fruit matures. Hardening occurs via secondary cell wall formation and lignification. The process of secondary wall formation in fruit tissues has not been studied to any great extent. However, based on the structural similarities between endocarp tissue and wood, information about this process can be inferred from studies on wood formation. In plant stems, xylem cells undergo a series of changes as they transform from fleshy to woody tissue. These include cell elongation, cell expansion, secondary cell wall deposition, programmed cell death, and finally heartwood formation (Dejardin et al., 2010). Secondary walls are comprised of multiple layers made up of cellulose, hemi-cellulose, and lignin with smaller amounts of pectin and proteins.

**FIGURE 4:**
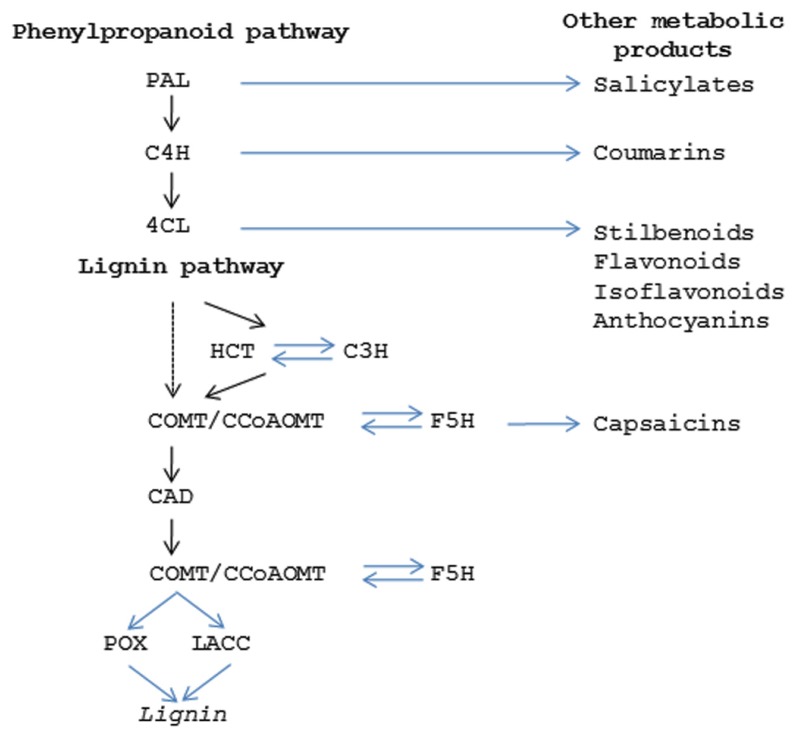
**Secondary metabolic pathways.** Diagram showing the enzymes in the phenylpropanoid pathway (PAL, phenylalanine ammonia lyase; C4H, cinnamic acid 4-hydroxylase; 4CL,4-coumarate:coenzyme A ligase) which produces the precursor products for lignin (HCT, hydroxycinnamoyl-CoA shikimate/quinate hydroxycinnamoyl transferase; C3H, 4-coumarate 3 –hydroxylase; COMT, caffeic acid O-methyltransferase; CCoAOMT, Caffeoyl CoA O-methyltransferase; F5H, ferulate 5-hydroxylase; CAD, cinnamyl alcohol dehydrogenase; POX, peroxidase; LACC, laccase). Steps in phenylpropanoid and ligin pathways that give rise to other secondary metabolism products are indicated.

Lignin provides a matrix within secondary cell walls for polymerization of cellulosic and hemi-cellulosic polymers which together contribute to providing tissue rigidity and tensile strength (Novaes et al., 2010). Most of the genes for the major enzymes in the pathway and the potential regulatory points have been identified (**Figure 4**; Boerjan et al., 2003). Lignin is formed from the phenylpropanoid pathway, the end products of which are coniferyl and sinapyl alcohols. These lignin monomers serve as the basis for lignification which is the process of producing the lignin polymer via oxidative reactions aided by peroxidases and laccases. Radical coupling of the monomers, particularly cross-coupling with the growing polymer, is a multi-step process that produces the complex lignin polymer.

The mechanism of endocarp hardening in peach has been investigated to a limited extent examining only one or two components or enzymes in the composition and formation of the stone tissue (Ryugo, 1963; Abeles and Biles, 1991; Alba et al., 2000; Hayama et al., 2006) Ryugo documented in the early 1960s that peach stones are rich in lignin, the seasonal pattern of lignin accumulation, and the presence of lignin biosynthesis intermediates (Ryugo, 1961, 1963). These studies and others have shown an increase in stone dry weight and lignification that begins in the second stage of fruit development until maturity (Ryugo, 1961; Nakano and Nakamura, 2002). More recently, biochemical analysis of drupes including olive, black walnut, peach, and coconut indicate they contain nearly twice as much lignin as wood, suggesting that the process of secondary wall formation can occur to a relatively extreme degree in fruit endocarp tissues (Mendu et al., 2011).

In addition to lignin, the phenylpropanoid pathway produces other secondary metabolic products that play important roles in fruit function (**Figure 4**). In some cases these compounds are critical for conferring seed protection and specifying seed dispersal. Coumarins, stilbenes, flavonols, and isoflavonoids have anti-microbial properties that limit bacterial and fungal disease (Dixon and Paiva, 1995). Other compounds contribute to fruit flavor and aroma; either attracting or deterring herbivores (Smith, 1982; Biggs and Northover, 1988; Peters and Constabel, 2002; Vom Endt et al., 2002). Herbivores are also strongly influenced by fruit coloration which is often attributable to anthocyanins and confer red or purple colorations. While this topic will not be extensively covered here, the fact that many of these functions arise from modifications of the same core enzymatic pathway highlights how relatively small changes in the control of secondary metabolic products can have large impacts on fruit phenotypes.

## GENETIC BASIS FOR ENDOCARP SPECIFICATION

Advances in genetics and genomics technologies are speeding identification of the underlying genes and signaling pathways that control differentiation of ovarian tissues into endocarp, mesocarp, and exocarp. *Arabidopsis* is leading the way and the information gained is now being translated to numerous other crops. While our current knowledge is still limited, it is becoming apparent that the same or very similar cellular programs contribute to pericarp tissue differentiation in a variety of species. Here, we review and discuss the developments regarding this emerging field of study in the Brassicaceae, Rosaceae, and Solanaceae families.

### BRASSICACEAE

Brassicaceae includes a number of economically important plants such as mustard, cabbage, radish, broccoli, and turnips. The model plant *Arabidopsis thaliana* is also a member of this family. Most Brassica species have a dehiscent pod-like fruit called a silique (long and narrow) or silicle (short and wide) and contain a distinctive replum tissue that separates the two valve margins. Mutagenesis screens in *Arabidopsis* have generated a large number of fruit morphology mutants. Some of these were found to contain defects in the dehiscence process and were named according to their phenotypes including *indehiscent* (*ind*), *shatterproof* (*shp*), *alcatraz* (*alc*), *spatula* (*spt*), *fruitfull* (*ful*), and *replumless* (*rpl*; reviewed by Dinneny and Yanofsky, 2004; Dinneny et al., 2005; Lewis et al., 2006). The identification and cloning of the underlying genes has provided insight into the molecular mechanisms of dehiscence and how pericarp tissues differentiate and lignify during development.

**FIGURE 5:**
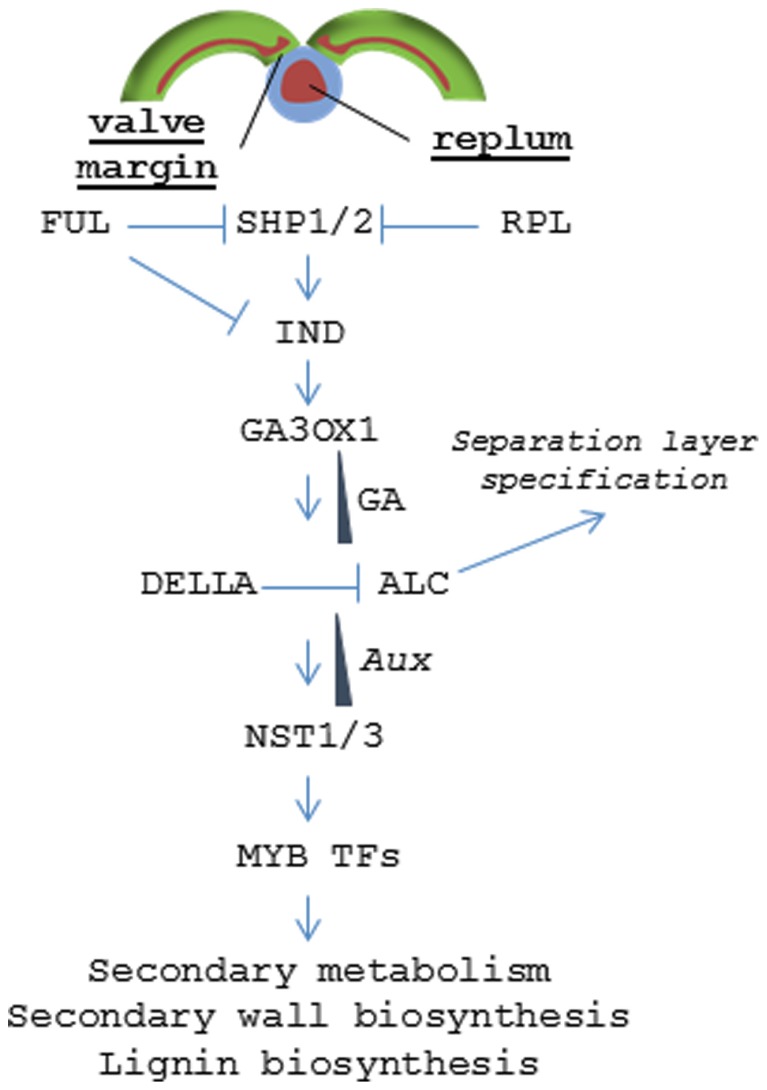
**Signaling pathway regulating dehiscence in *Arabidopsis*.** Model depicting the hierarchy of transcription factors that control tissue patterning in the valve margin and replum.

Specific zones within the pericarp are controlled by a coordinated set of transcription factors (TF) that specify tissue fate (**Figure 5**). The lignified valve margin layer responsible for pod shatter is determined by two partially redundant genes, *SHP1* and *SHP2*, which encode MADS-BOX TFs. *SHP* is closely related to the class C gene *Agamous* (*AG*) that regulates flower carpel and stamen identity (Liljegren et al., 2000). Siliques in *shp1*/*shp2* double mutants do not lignify within the valve margin layer and fail to dehisce. Specification of valve cell fate by *SHP1* and *SHP2* is delimited by another MADS-BOX TF called FUL. *FUL* is expressed throughout the valves and negatively regulates *SHP1* and *SHP2*, restricting their activity to the valve margin. Arabidopsis *ful* mutants produce siliques where the entire valve mesocarp lignifies while FUL over-expression leads to conversion of valve margins and outer replum into non-lignified valve tissue; resulting in indehiscent siliques (Ferrándiz et al., 2000). SHP1 and SHP2 positively regulate a basic helix loop helix (bHLH) TF called IND (Liljegren et al., 2004). *IND* is also negatively regulated by FUL and has been shown to prevent valve margin cells from adopting a valve identity (Girin et al., 2010). It does this by coordinating an auxin gradient in the separation layer cells resulting in the formation of lignified valve margin tissues required for valve separation (Sorefan et al., 2009).

The non-lignified valve margin layer is determined by ALC and SPT, two partially redundant bHLH class TFs (Rajani and Sundaresan, 2001; Groszmann et al., 2011). *alc* mutants develop relatively normal siliques but lack the non-lignified layer (separation layer) that separates the lignified valve margin from the replum lignification zone. This blocks valve separation resulting in an indehiscent phenotype. ALC is negatively regulated by gibberellic acid (GA) through the DELLA repressor. IND induces expression of a gene encoding a GA activating enzyme (*GA3OX1*) resulting in GA accumulation in the separation layer and subsequent dissociation of the DELLA protein from ALC (Arnaud et al., 2010).

In the replum, RPL serves a similar function as FUL and prevents those non-lignifying cells from adopting a valve margin cell fate by inhibiting *SHP2* expression in the replum (Roeder et al., 2003). In the more severe *rpl* mutant phenotypes, the lignified valve margin layer intrudes into the replum lignification layer resulting in a partially indehiscent silique. *RPL* encodes a BELL1 family homeodomain TF (Roeder et al., 2003). BELL1 had been previously known to negatively regulate *AG* (Yanofsky et al., 1990; Western and Haughn, 1999).

SHP1 and SHP2 have retained some class C function and are marginally redundant with AG in ovule and floral organ differentiation and appear to be regulated by AG (Pinyopich et al., 2003). Ectopic expression of SHP resulted in conversion of sepals to carpel-like structures even in the absence of *AG* (Favaro et al., 2003). Thus, sub-functionalization of *AG* genes has resulted in overlapping and partially redundant pathways controlling different stages of flower and fruit development (Savidge et al., 1995; Colombo et al., 2010). This is also evident for *STK*, another *AG*-like MADS-BOX gene that resides in a distinct clade. STK controls funiculus development and seed release and shares partial redundancy with AG, SHP1 and SHP2 in specifying ovule cell fate but does not have a class C function (Pinyopich et al., 2003; Zahn et al., 2006). Still, STK has retained the capacity for class C function as ectopic STK expression can complement carpel formation in an *agamous* mutant (Favaro et al., 2003). All four members of the AG lineage are known to function in multi-meric MADS-BOX protein complexes with proteins encoded by members of the *SEPALLATA* (*SEP*) floral organ identity genes that together modulate downstream transcriptional activation (Davies et al., 1996; Pelaz et al., 2000; Favaro et al., 2003). The finding that *AG*-like genes independently control both dehiscence and seed release imply that this closely related family of transcriptional regulators has evolved to control distinct fruit development processes (Pinyopich et al., 2003).

While the mechanisms regarding valve margin and replum specification are known in Arabidopsis, signaling associated with en*b* determination is less clear. Each of the dehiscence mutants *shp1*, *shp2*, *ind*, *alc*, *ful*, and *rpl* show relatively normal endocarp development with the exception of the quintuple mutant *ind alc shp1 shp2 ful* that displays a complete loss of pericarp lignification (Liljegren et al., 2004). This finding suggests that en*b* cell fate requires this same pathway but there may be significant redundancy and/or signaling feedback loops that are not fully understood. But once pericarp tissue identity has been established, at least one downstream pathway leading to tissue differentiation and lignification is known. Two NAC (NO APICAL MERISTEM) family TFs called NST1 and NST3 (NO SECONDARY WALL THICKENING) [also known as SND (SECONDARY WALL-ASSOCIATED NAC DOMAIN)] were found to regulate secondary wall formation and lignification within the endocarp layers (Mitsuda and Ohme-Takagia, 2008). *nst1 nst3* double mutants show little or no lignin accumulation and were found to be required for expression of genes involved in cell wall biosynthesis and secondary metabolism (Mitsuda and Ohme-Takagia, 2008). *NST1* was also shown to regulate anther dehiscence and lignification of woody and vascular tissues (Mitsuda et al., 2005; Zhong et al., 2007). NST1 acts upstream of a series of MYB (myeloblastosis) TFs that, in turn, directly regulate the expression of genes encoding key enzymes in the phenylpropanoid pathway that drives lignin biosynthesis (Mitsuda et al., 2007; Zhong et al., 2007, 2008). Orthologs of *NST1* have similar functions in *Medicago* and poplar vascular tissues; suggesting that lignification in endocarp tissues occurs via the same pathway as that in vegetative tissues and wood (Zhao et al., 2010; Zhong et al., 2010). Still, there is a gap in our understanding of how *NST1* and *NST3* become activated in a tissue specific fashion. The finding that *IND* controls auxin patterning may hold the key as wood formation is also known to be regulated by the establishment of local auxin gradients (Nilsson et al., 2008; Sorefan et al., 2009).

Based on the knowledge gained in Arabidopsis, a number of researchers have evaluated whether these same genetic pathways are conserved in other Brassica species. The valve margins of dehiscent fruit in *Lepidium campestre* are very similar to that of Arabidopsis and expression of *ALC*, *IND*, *SHP1*, and *SHP2* was likewise found to be limited to the valve margins (Mummenhoff et al., 2009). Lenser and Theißen (2013) showed that RNAi knock-down or over-expression of *IND*, *ALC*, *SHP*, or *FUL* resulted in the anticipated indehiscent phenotypes and mimicked those observed in Arabidopsis with only minor differences. The regulatory interactions among these genes in *FUL* or *IND* lines were also conserved as ALC was found to be a negative regulator of *IND* in both *L. campestre* and Arabidopsis (Lenser and Theißen, 2013). In contrast, gene expression of *ALC*, *IND*, *SHP1*, and *SHP2* was found to be abolished in the tissue corresponding to the valve margins in *Lepidium appelianum*, a Brassica species that produces an indehiscent fruit lacking the separation layer (Mummenhoff et al., 2009). The authors concluded that the evolution of indehiscence in this species likely involved changes in an upstream regulator of the pathway. Expression and functional studies ruled out known regulators including orthologs of *FUL*, *RPL*, and *APETALA2* (*AP2*; Mühlhausen et al., 2013). Some dehiscent *Brassica* species vary with respect to the development of valve margins. *Erucaria erucarioides* and *Cakile lanceolata* produce heteroarthrocarpic fruits where only the proximal segment of the silique dehisces while the distal portion remains indehiscent (Avino et al., 2012). Expression of the valve margin identify genes *ALC*, *FUL*, *IND*, *RPL*, *SHP1*, and *SHP2* was largely conserved in the proximal dehiscent part of the fruit but absent in the distal indehiscent portion. Collectively, these studies indicate that evolutionary adaptations in *Brassica* siliques are, in part, driven by changes in the expression of a single coordinated developmental pathway that helps define the valve, valve margin, separation layer, and replum lignification zones.

### ROSACEAE

**FIGURE 6:**
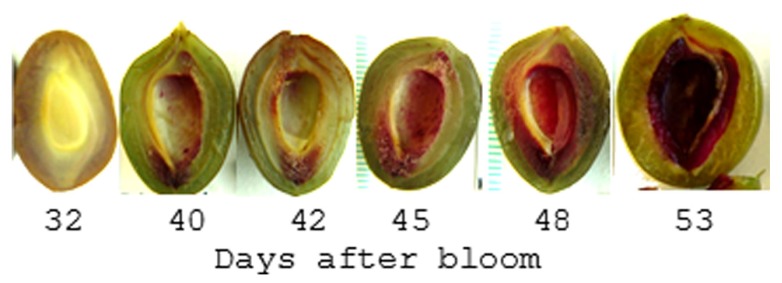
**Pattern of lignin production in plum endocarp.** Shown is a plum fruit series sectioned parallel to the suture line and stained with Phloroglucinol-HCL which turns red in the presence of lignin. After 53 days the endocarp begins to harden such that it can no longer be cut with a scalpel.

In contrast to the Brassicaceae, plants in the family Rosaceae encompass an extremely wide range of fruiting types including drupes, pomes, achenes, as well as a number of dry dehiscent and indehiscent fruits. The genus *Prunus* exclusively contains drupes including peaches, plums, apricots, almonds, and cherries which produce a large lignified endocarp that surrounds the seed; commonly called the stone. These fruits grow in a sigmoidal pattern and display a pause in growth that coincides with endocarp hardening. This may be a consequence of the increased carbon and energy demands associated with lignification (Callahan et al., 2009). Recent studies on the pattern and timing of endocarp lignification reveal it is a highly coordinated process that occurs over a 2- to 3-week period (Tani et al., 2007; Dardick et al., 2010; Hu et al., 2011; Lombardo et al., 2011; **Figure 6**). While the timing can vary between cultivars, lignin is often first detectable approximately 35-45 days after bloom in a thin endocarp layer along the fruit suture and in the funiculus. But after several days the entire endocarp begins to lignify. Hardening appears to follow the same pattern as lignin accumulation since the tissue in which lignin is first detectable is also the first to harden.

**FIGURE 7:**
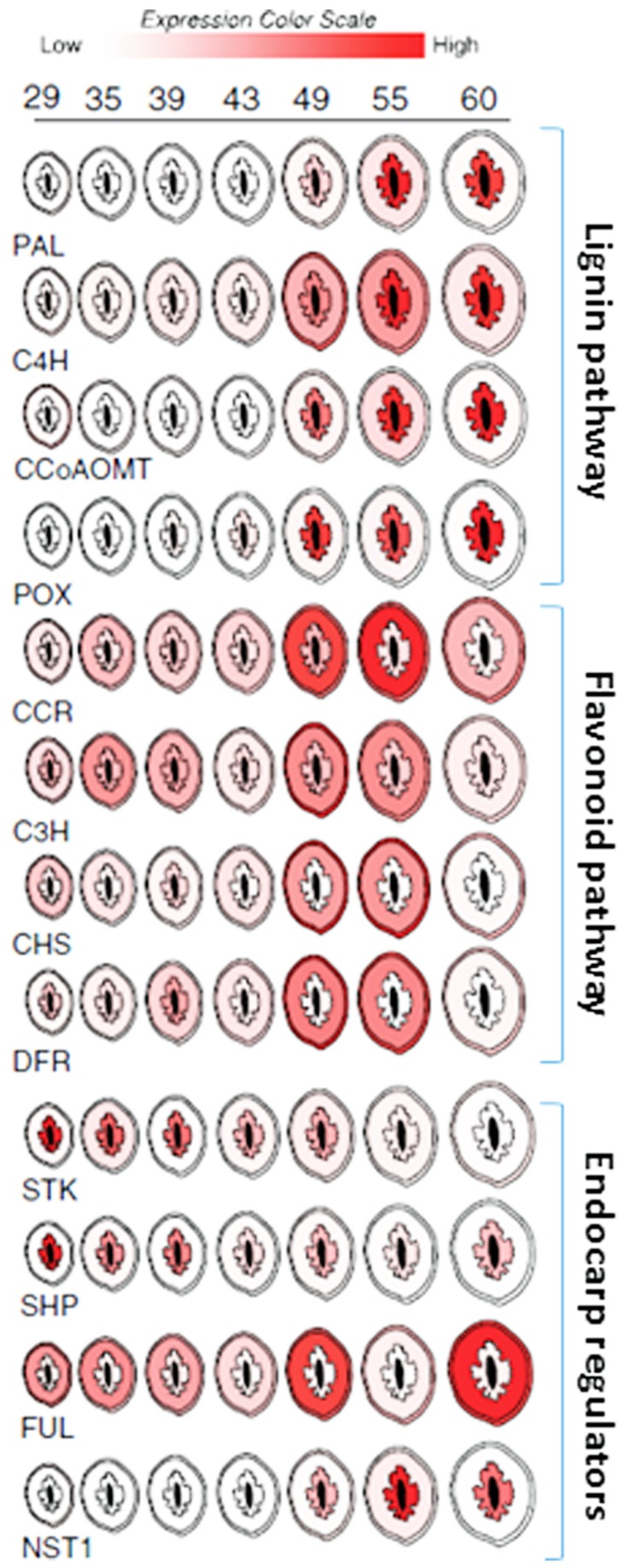
**Spatial/temporal pattern of gene expression in developing peach fruit.** Cross sections of peach fruits from 29 to 60 days after bloom are depicted. Relative gene expression levels within the exocarp (outer skin), mesocarp (fleshy middle), and endocarp (inner stone) sections are color coded (scale bar at top). Expression in seed (black center) is not shown. Target gene abbreviations are listed below each series and the relevant pathways are delimited by brackets and labeled (right).

While functional studies are still lacking, expression profiling data suggests that many of the same genes that control dehiscence in Brassica species also control endocarp development in peach (*Prunus persica*; Dardick et al., 2010; **Figure 7**). The peach homologs of *SHP* and *STK* were found to be up-regulated in the endocarp shortly after pollination. *SHP* and *STK* expression were restricted to the endocarp and seed but gradually decline near the onset of lignin accumulation. Likewise, *FUL* expression remained higher in the mesocarp and exocarp but was constitutively low in the endocarp. This is consistent with a possible role in delimiting endocarp lignification margins. Upon the decline of *SHP* and *STK*, the expression of a peach *NST1* homolog rapidly accumulated along with secondary metabolism and cell wall biosynthesis genes. While clear homologs of *ALC* and *IND* were not found in peach, the two most similar genes were not endocarp specific (Dardick et al., 2010). *ALC* was previously shown to be specific to *Brassica* species and evolved as a recent duplication of another bHLH TF called *SPATULA* (*SPT*; Groszmann et al., 2008). Tani et al., 2011 showed that the expression patterns of peach *SPT* were consistent with a role in specifying endocarp margins. Collectively, these data imply that highly similar pathways likely control pericarp development in both *Prunus* and Brassica fruits.

Peach mesocarp and exocarp tissues accumulate other secondary metabolic compounds including flavonoids. Flavonoids are an important class of compounds found in nearly all fruit. They provide resistance against disease and pests and contribute to fruit flavor and color. Well known examples include the anthocyanins which are commonly responsible for the orange, red, and purple colorations found in many fruits. Like lignin, flavonoids are also synthesized via secondary metabolism pathways which are thought to be competitive with lignin since both draw on the same precursors of the phenylpropanoid pathway. Peach fruit showed simultaneous activation of the lignin and flavonoid pathways during early fruit development (Dardick et al., 2010; Hu et al., 2011). These events were spatially coordinated such that phenylpropanoid pathway genes were induced in all three pericarp layers; endocarp, mesocarp, and exocarp (though to a much greater degree in endocarp). But in the endocarp this upregulation was accompanied by lignin pathway induction and concomitant flavonoid pathway repression while in the mesocarp and exocarp flavonoid pathway genes were induced and lignin genes were repressed (**Figure 7**). Presumably this coordination allows the fruit to accumulate defense compounds, flavor, and color development in the mesocarp and exocarp while simultaneously enabling endocarp lignification. Thus, seed protection via endocarp lignification appears to be coordinated with the production of compounds necessary for defense, herbivore attraction, and seed dispersal.

There is tremendous variation in *Prunus* endocarp phenotypes which have been selected through breeding. For example, almond shells vary with respect to endocarp thickness, hardness, and brittleness. These agronomic qualities are critical for processing almonds and other types of nuts. Some peach varieties suffer from a phenotype called “split pit” where the endocarp does not seal along the suture leaving the seed vulnerable to pests and disease. Peach cultivars that resume rapid fruit growth before the stone has completely hardened are more likely to have split pits. Tani et al. (2007) found that SHP expression in a split pit resistant variety was lower during the lignification stage while FUL expression was significantly elevated in the sensitive variety during later stages of fruit growth.

**FIGURE 8:**
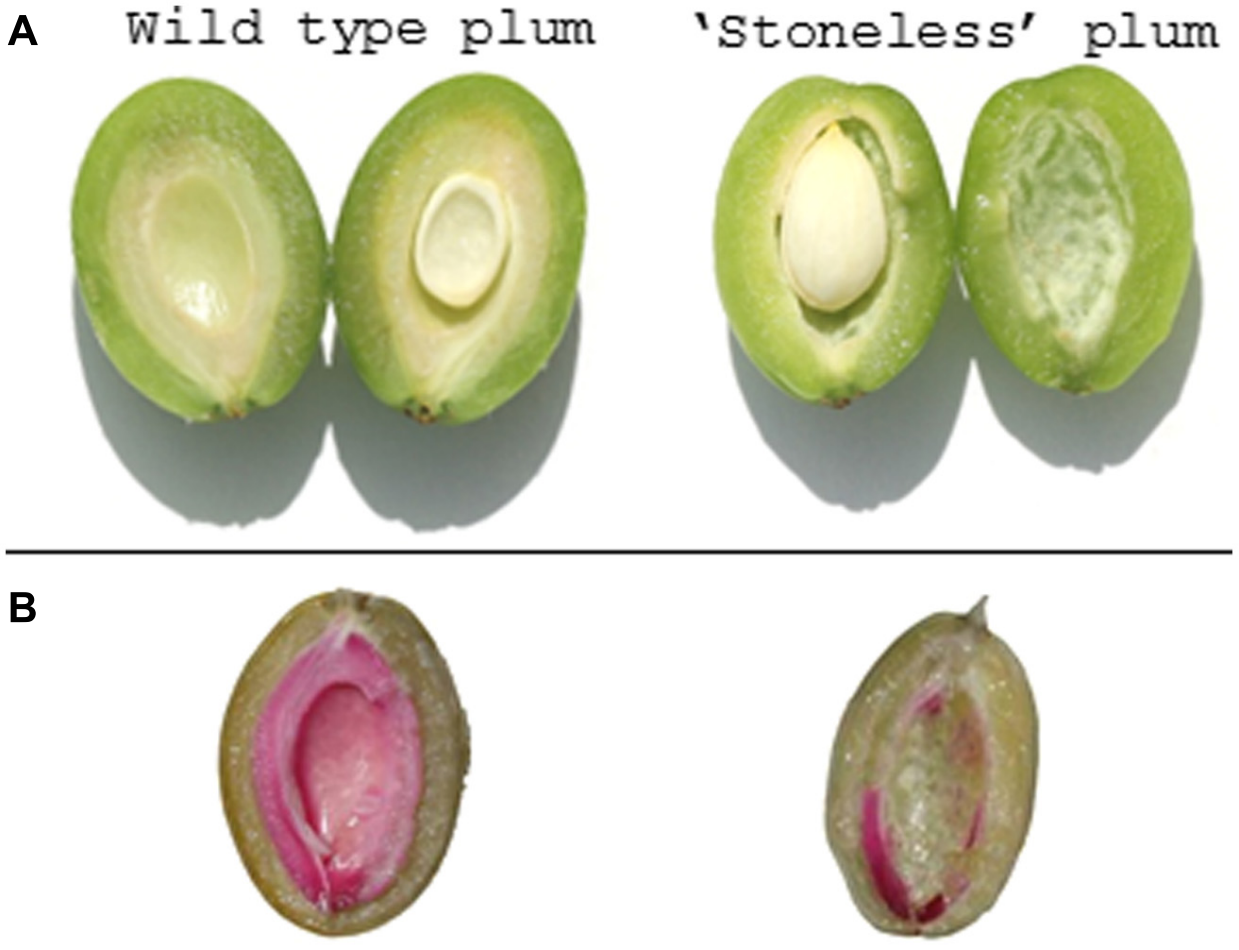
**Naturally occurring *prunus* mutant phenotype. (A)** Transverse section of a wild-type plum (left) and “Stoneless” (right). The endocarp is visible as a whitish tissue layer surrounding the seed. In “Stoneless” a cavity is visible where endocarp tissue is normally found. **(B)** Same fruit stained with phloroglucinol-HCL to visualize lignin production (red color). Little or no lignin staining is found in “Stoneless.”

“Stoneless” is a naturally occurring phenotype first found in a wild-type plum (*Prunus domestica*) species from France, Sans Noyau (Callahan et al., 2009). “Stoneless” does not completely develop the endocarp layer resulting in a partially naked seed that sits within an empty fruit cavity (**Figure 8**). We have observed that the “Stoneless” phenotype is strongly influenced by the environment since in years with hot spring temperatures fruit tend to contain a more complete stone while in cooler years very little stone is present (Callahan et al., 2009). The hardened tissue that remains in “Stoneless” appears to coincide with the funiculus and a portion of the plancental endocarp wall (Callahan et al., 2009). Expression studies show that the lignification process likely functions normally in “Stoneless” since secondary metabolism genes are still induced. The lack of endocarp tissue suggests that this mutant does not contain a complete endocarp layer.

A handful of studies have also been carried out in other Rosaceous genera. Hawthorn (*Crataegus* spp.) produces a pome fruit that often contains a hardened endocarp like a drupe, however, some species are known to produce soft, edible endocarps. Expression profiling studies revealed that unlike that observed in those with a hardened endocarp, the lignin pathway was not upregulated in the endocarp of soft hawthorns (Dai et al., 2013). In another pome fruit, japanese pears (*Pyrus pyrifolia*), examination of the gene expression patterns of *SHP* and *FUL* among numerous other MADS-box genes showed that *SHP* expression was limited to the fruit core during early fruit development and was largely absent in the fruit cortex and skin, consistent with the ovarian origin of the core (Ubi et al., 2013). In contrast, *FUL* expression was more uniform and was present in skin, cortex, and core regions. While the pear core itself does not lignify, the adjoining layer is lined by disorganized stone cells or schlereids that can be found scattered throughout the hypanthium resulting in a gritty flesh texture (Tao et al., 2009). In strawberry (*Fragaria ananassa*), Daminato et al. (2013) found that silencing or over-expression of *SHP* did not appreciably alter fruit form. This may be expected since the flesh of strawberry is derived from the flower receptacle and not the pericarp. However, *SHP* transgenic lines did show significant changes in ripening time. These results are consistent with similar experiments in tomato where *SHP* was also shown to be a key regulator of fruit ripening (Itkin et al., 2009). Due to availability of whole genome sequences for a number of Rosaceous species including strawberry, apple, and peach along with established transformation systems, this family offers an excellent opportunity to further study the diversification of fruit development (Velasco et al., 2010; Shulaev et al., 2011; Verde et al., 2013).

### SOLANACEAE

The family Solanaceae also contains a wide variety of both dry and fleshy fruit types which have repeatedly undergone a number of berry-to-capsule and capsule-to-berry transitions. A detailed developmental analysis by Pabón-Mora and Litt (2011) showed that early developmental stages are similar among capsular and berry type fruits. Later developmental stages were marked by differentiation of endocarp including changes in cell number, cell expansion, and sclerification.

Tomato (*Solanum lycopersicum*) has long served as a model for fleshy fruit development and ripening. The role of a tomato *SHP* homolog called *TOMATO AGAMOUS-LIKE 1* (*TAGL1*) has been extensively studied (Vrebalov et al., 2009; Itkin et al., 2009). Silencing of *TAGL1* resulted in both a thinner pericarp layer and impaired ripening. Pericarp thickness was reduced by approximately 50% in *TAGL1* silenced lines compared to wild-type which was attributed to fewer numbers of cell layers (Vrebalov et al., 2009). This same effect on pericarp thickness was not observed in the small fruited MicroTom tomato variety which has a naturally thin pericarp (Pan et al., 2010). Ripening in *TAGL1* silenced lines or lines expressing a chimeric dominant TAGL1 repressor displayed reduced carotenoids (the pigments responsible for fruit coloration in tomato), lower levels of ethylene, and repression of ripening associated genes including those associated with ethylene biosynthesis and signaling (Itkin et al., 2009; Vrebalov et al., 2009; Pan et al., 2010). The role of *TAGL1* in ripening was distinct from the previously described MADS-BOX gene *RIPENING INHIBITOR* (*RIN*), however, these two MADS-BOX genes may overlap in their ability to induce ethylene as TAGL1 protein was shown to bind the *ACC synthase2* (*ASC2*) promoter in a transient assay (Itkin et al., 2009). In contrast, TAGL1 over-expression led to increased fruit fleshiness, fruit-like sepals that ripened, and increased accumulation of carotenoids (Itkin et al., 2009; Vrebalov et al., 2009). When transformed into an *Arabidopsis shp1 shp2* double mutant, *TAGL1* did not rescue the indehiscent phenotype suggesting that *TAGL1* and *SHP1* may have functionally diverged. In contrast, over-expression of the peach *SHP* homolog (also called *PpPLENA*) in tomato gave rise to a phenotype reminiscent of that observed for *TAGL1* (Tadiello et al., 2009). Experiments to test whether peach *SHP* can complement *Arabidopsis* mutants have not yet been reported. It was shown, however, that ectopic expression of *SHP* derived from the Rosaceous species *Taihangia rupestris* led to conversion of sepals to carpelloid structures and promoted premature pod shatter (Lü et al., 2007). Collectively, these findings suggest that *SHP*-like genes have conserved functions but may have differentiated during the evolution of new fruiting structures and seed dispersal strategies.

Tobacco species are also members of the Solanaceae and produce dry capsular fruits that dehisce upon maturation. Over-expression of a *Nicotiana tobacum* homolog of *FUL* led to indehiscent phenotypes in both *N. tobacum* and *N. sylvestris* that was attributed to reduced lignification along the carpel midrib (Smykal et al., 2007). Knock down of *SHP* through virus induced gene silencing (VIGS) led to a complete loss of dehiscence and lack of lignified layers lining the dehiscence zones (Fourquin and Ferrándiz, 2012). In addition, *SHP* silencing caused significant alterations in flower development marked by incomplete carpel fusion and shortened styles. Meristem specification was also altered leading to additional carpel and stamen abnormalities. Simultaneous silencing of *SHP* and *AG* led to further loss of stamen and carpel identity as did silencing *AG* alone, suggesting that *SHP* plays only a minor role in C-function (Fourquin and Ferrándiz, 2012). The data imply a limited sub-functionalization of *SHP* from the progenitor C-class TF *AG* in *N. benthamiana*. Similar C-function overlap between *AG* homologs in snapdragon (called *FARINELLI* and *PLENA*, respectively) and in Petunia (called *PETUNIA MADS-BOX GENE 3* (*PMADS3*) and *FLORAL BINDING PROTEIN 6* (FBP6)) was also observed (Causier et al., 2005; Heijmans et al., 2012).

## BROADER PERSPECTIVES

As our knowledge of fruit development expands beyond model crops, some of the genes responsible for natural variation in fruit forms are beginning to emerge. A recent report on *Medicago* showed that the coiled pod morphology unique to some members of that genus was likely the result of amino acid changes with a *SHP* homolog that promotes increased valve margin lignification (Fourquin et al., 2013). The loss of the hardened endocarp in commercial oil palm varieties was recently traced to mutations in the DNA binding domain of SHELL, a *STK* homolog, that were shown to prevent association with SEP (Singh et al., 2013). This stands in contrast to Arabidopsis where *STK* does not appear to play a role in endocarp differentiation (Pinyopich et al., 2003; Zahn et al., 2006). Findings such as these provide our first glimpse into how plants have evolved such a dizzying array of fruiting structures and seed dispersal strategies. It is now becoming clear that rapid conversions of fruit form and function are possible through changes in the expression patterns and/or activity of sub-functionalized *AG*-like genes or their associated regulators. These changes can lead to spatial/temporal shifts in cell fate determination accompanied by modifications in secondary metabolic activities that mediate downstream events such as lignification, coloration, and/or generation of herbivore attractants/repellents.

The current emphasis on Arabidopsis as a model system has undoubtedly introduced some level of bias into our current level of knowledge and there is a clear need for plant biologists to expand molecular developmental studies to other crops. For example, the degree to which *AG*-like genes and their known partners have played a role in natural selection of plant species remains to be seen. New sequencing technologies that enable gene mapping through genome-wide association studies (GWAS) along with a growing genomic toolkit promise to address these questions. Ongoing experiments to unveil the specific changes that have allowed different fruit forms to emerge within the same plant lineage will help shed light on the identity of key developmental pathways, the degree of plasticity of these regulatory systems, and how specific plants have adapted to occupy new niches.

## Conflict of Interest Statement

The authors declare that the research was conducted in the absence of any commercial or financial relationships that could be construed as a potential conflict of interest.
